# Improving Post-Hospitalization Transition Outcomes through Accessible Health Information Technology and Caregiver Support: Protocol for a Randomized Controlled Trial

**DOI:** 10.4172/2167-0870.1000240

**Published:** 2015-10-03

**Authors:** John D Piette, Dana Striplin, Nicolle Marinec, Jenny Chen, Lynn A Gregory, Denise L Sumerlin, Angela M DeSantis, Carolyn Gibson, Ingrid Crause, Marylena Rouse, James E Aikens

**Affiliations:** 1 Ann Arbor Department of Veterans Affairs Center for Clinical Management Research, Ann Arbor, Michigan, USA; 2 Department of Health Behavior and Health Education, School of Public Health, University of Michigan, Ann Arbor, Michigan, USA; 3 Department of Internal Medicine, School of Medicine, University of Michigan, Ann Arbor, Michigan, USA; 4 Department of Family Medicine, University of Michigan, Ann Arbor, Michigan, USA

## Abstract

**Objective:**

The goal of this trial is to evaluate a novel intervention designed to improve post-hospitalization support for older adults with chronic conditions via: ***(a)*** direct tailored communication to patients using regular automated calls post discharge, ***(b)*** support for informal caregivers outside of the patient’s household via structured automated feedback about the patient’s status plus advice about how caregivers can help, and ***(c)*** support for care management including a web-based disease management tool and alerts about potential problems.

**Methods:**

846 older adults with common chronic conditions are being identified upon hospital admission. Patients are asked to identify a “CarePartner” (CP) living outside their household, i.e., an adult child or other social network member willing to play an active role in their post-discharge transition support. Patient-CP pairs are randomized to the intervention or usual care. Intervention patients receive automated assessment and behavior change calls, and their CPs receives structured feedback and advice via email and automated calls following each assessment. Clinical teams have access to assessment results via the web and receive automated reports about urgent health problems. Patients complete surveys at baseline, 30 days, and 90 days post discharge; utilization data is obtained from hospital records. CPs, other caregivers, and clinicians are interviewed to evaluate intervention effects on processes of self-care support, caregiver stress and communication, and the intervention’s potential for broader implementation. The primary outcome is 30-day readmission rates; other outcomes measured at 30 days and 90 days include functional status, self-care behaviors, and mortality risk.

**Conclusion:**

This trial uses accessible health technologies and coordinated communication among informal caregivers and clinicians to fill the growing gap between what discharged patients need and available resources. A unique feature of the intervention is the provision of transition support not only for patients but also for their informal caregivers.

## Introduction

### The causes of unsuccessful post-hospitalization transitions for chronically-ill patients

Between 20% and 30% of hospitalized medical patients are readmitted within the first 30 days post-discharge [1–4]. Adverse outcomes often result from patients’ difficulty managing their complex self-care regimens [5,6]. Problems include inadequate clinician monitoring post discharge [2,7,8], and caregivers often are left out of the communication loop further contributing to preventable rehospitalization, emergency care visits, and higher healthcare costs [9–11]. Care management can reduce rehospitalization rates and mortality risk [12–14], but resources required to provide these services are often unavailable [15]. Nurse telephone calls post-discharge are labor intensive [16,17], and evidence is still weak that post-discharge clinician follow-up alone improves outcomes [18–24]. Comprehensive discharge planning is a key feature of transition support programs [25,26], but is frequently impossible to provide during brief hospital stays and also has variable evidence as a stand-alone intervention [27]. Patients often do not understand their medications or chief diagnosis at discharge [28], and few systems are in place to ensure that key information is transferred to the clinicians who will follow the patient in ambulatory care [29–31].

### The importance of strengthening informal care post hospitalization

Informal caregivers often play an important role in supporting chronically-ill patients’ efforts to: follow self-management plans, identify early warning signs of acute illness, absorb the extensive self-care information patients need to stay well [32–35], use formal health systems effectively, and cope emotionally with their ongoing illness [36–38]. Unfortunately, social networks of potential caregivers are often fragmented [39], and patients frequently are unwilling to ask loved ones to play a more active role [40]. Although adult children typically feel involved in their ill parents’ care regardless of geographic distance [41], there typically is no internal mechanism within the patient’s social context for these potential caregivers to assume clearly defined tasks [42,43].

### Interactive voice response (IVR) telephonic communication to improve transitions

In this trial, we use IVR calls as a primary tool for increasing communication between patients, informal caregivers, and clinicians. IVR communication has low marginal cost and is accessible to patients regardless of their location or computer literacy [44,45]. Automated calls can identify important health concerns arising post-discharge [7,46], and studies show that most patients are very satisfied with such services; some even preferring automated calls to “live” calls from a clinician [47–51]. Chronic disease management models that include IVR calls are feasible [48,52,53] and provide valid and reliable data about patients’ status between visits [52]. In prior trials we have shown that disease management programs including automated telephone assessments can improve outcomes for outpatients with diabetes or with heart failure [54–58].

The current trial uses IVR-based caregiver involvement to support the goals of transition support as defined in project “BOOST” (better outcomes for older adults through safe transitions; Table 1) [26]. In brief, this model includes pre- and post-discharge components supported by specific recommendations and structured clinical forms for managing key patient-centered information, e.g., a patient discharge preparation list, Personal Health Record, and forms for reconciling medications. A quasi-experimental trial of the BOOST service showed a 50% reduction in 30-day rehospitalization rates and a 57% reduction at 90 days [26]; results were similar in a subsequent randomized trial [59]. Several recent studies have demonstrated that feedback to informal caregivers may increase the effectiveness of IVR-based interventions supporting self-management while simultaneously decreasing caregiver distress [58,60–62].

### Intervention and conceptual framework

**Figure 1:**
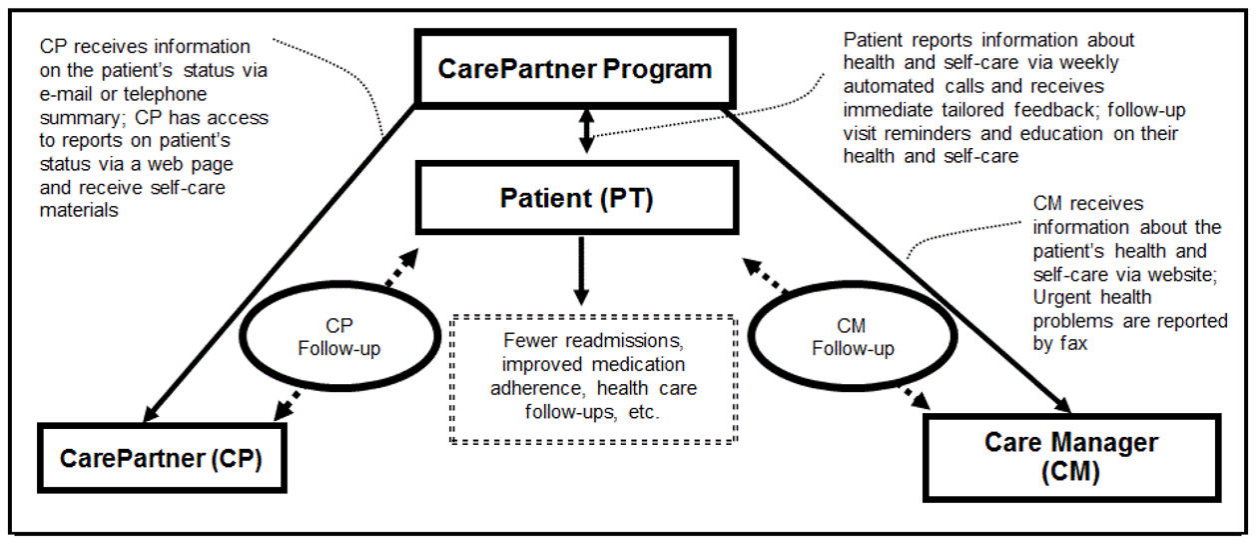
CarePartner program mechanism of action.

The intervention evaluated in the current trial is designed to improve outcomes through three separate mechanisms of action (Figure 1): tailored and timely education provided directly to the patient via IVR, improved clinical follow-up based on monitoring reports and fax alerts, and more active and empowered caregivers. To our knowledge this is the first model to improve the quality and quantity of informal care for discharged patients by: (a) increasing informal caregivers’ knowledge of patients’ health status and behavioral needs, (b) educating caregivers about the key goals of successful community transitions, and (c) giving caregivers targeted advice about how to address problems and communicate effectively.

## Methods

### Recruitment

Patients are identified at the time of an acute-care medical admission to: the University of Michigan Health System, the VA Ann Arbor Healthcare System, and a third non-profit private healthcare system in Michigan. Patients are eligible if they are at least 50 years of age and have a common chronic illness frequently associated with an increased risk of rehospitalization due to inadequate transition support [59], i.e., chronic heart failure, stroke, coronary artery disease requiring hospitalization, arrhythmia, chronic obstructive pulmonary disease, peripheral vascular disease, deep vein thrombosis, pulmonary embolism, pneumonia, type 2 diabetes, urinary tract infection, gastroenteritis, *Clostridium difficile* infection, or asthma. Patients are excluded if they: have a serious mental illness such as schizophrenia, are in hospice care, do not speak English, are unable to use a telephone, have a non-health system-affiliated primary care provider, are unable to nominate a potentially eligible “CarePartner” (see criteria below), or are cognitively impaired during hospitalization as determined by a validated screener [63]. Recruitment nurses use specially designed algorithms to conduct queries of admission data to identify potentially eligible patients’ diagnoses and possible exclusionary criteria. Additional patients are identified based on reviews of charts for newly admitted patients. The recruiter meets with potential recruits in the hospital to describe the study, screen for eligibility, and obtain informed consent.

Patients in both study arms are asked to nominate an informal caregiver or CarePartner (CP) living outside of their household who is willing to play a structured role in their transition care. We use the Norbeck Social Support Questionnaire (NSSQ) [64] to identify the people with whom the patient has the most frequent contact and who provide the most instrumental and emotional support. Based on NSSQ scores, research assistants assist the patient in identifying the most suitable person to serve as their CP. Potential CPs are contacted by the recruitment nurse in person or by phone during the hospital stay or when absolutely necessary, during the first 7 days post discharge. CPs are ineligible if they have a serious mental illness, do not speak English, are less than 21 years of age, or do not have access to email.

### Randomization

After patients complete their informed consent and baseline assessment, they and their CP are randomized to the intervention group or usual care. The randomization procedure is concealed to recruiters by means of pre-sealed envelopes. Group assignment is not disclosed to patients until after they have completed baseline surveys.

### Interventions

Usual care: All study participants continue to receive usual care in which discharge instructions and a written list of medications are presented by a member of the nursing staff. CPs randomized to the usual care group receive written information about how to successfully support the patient’s transition from hospital to home, including information provided by the national caregiver alliance (www.caregiver.org). They also receive self-care information specific to their patient-partner’s discharge diagnoses.

CarePartner program intervention: (Table 2) Intervention patients review with the recruitment nurse materials based on the BOOST transition program, such as the Personal Health Record and structured discharge preparation checklist. We use information from the inpatient record along with any specific instructions from the practice to identify each patient’s “care manager,” i.e., primary contact for post-discharge follow-up, prior to discharge. Also prior to discharge, patients have post-discharge primary care visits scheduled by the inpatient team with support from the recruitment nurse. Visit information is recorded in the patient’s Personal Health Record, and the recruitment nurse enters the dates/times into the IVR follow-up system so that patients and their CPs can receive reminder calls.

At the time of enrollment, intervention-group patients indicate the times and phone numbers with which they would like to receive their IVR assessment and self-care support calls. During the initial two weeks post discharge, patients receive daily assessment calls with up to three attempts made at 30-minute intervals. The system re-calls the patient if a busy signal is received, and if someone other than the patient answers the call, the service instructs the person to bring the patient to the phone or can call back at a later time. After the initial post-discharge period, patients receive IVR assessments three times per week for two weeks, and then weekly for the remainder of the 90 days. Calls to patients include statements and queries recorded in a human voice. Patients respond to requests for information using the touch-tone keypad on their telephone. Reminder messages to patients (via automated calls) and to their CPs (via email and automated reminder calls) reinforce dates of follow-up appointments and possible ways to address barriers such as transportation problems.

During IVR calls, patients receive recorded feedback about problems they report. Feedback messages are designed to: reinforce the importance of medication adherence, remind patients to use the information from their patient-centered record to schedule and attend appointments; remind patients about primary care and specialist follow-up; and teach patients various “red-flags,” i.e., indicators of worsening health status and how those should be addressed. Although most of the call content focuses on general self-care issues such as medication adherence, key disease-specific issues also are addressed. For example, for patients diagnosed with heart failure, the calling system asks patients about possible changes in weight and provides information about the connections between fluid retention and medication adherence, salt intake and fluid intake [65]. Action suggestions specify whether the patient should contact their CP and/or care manager and the suggested timeframe for responding. During each call, patients are instructed to call 911 if they are experiencing urgent problems such as chest discomfort. At the end of each call, patients have the option of hearing the name and phone number of their care manager.

Urgent problems such as breathing difficulties generate a fax alert. The thresholds for these alerts were negotiated with representatives of the primary care leadership in the participating health systems, and in some cases care managers are able to tailor those thresholds across patients and over time for a given patient. Care managers have access to a website that includes panel-level data based on patients’ most recent assessments that identifies patients with urgent and non-urgent problems. Care managers also are able to review patients’ complete calling history, including preferred calling times, rates of completing assessments, and what patients reported in each assessment call.

CPs can access feedback about the patient’s status via the Internet or a specially designed voicemail system. CPs receive e-mail reports summarizing the results of patients’ automated assessment and behavior change calls. Email feedback about urgent issues is accompanied by a brief automated call, alerting the CP to check their e-mail for a detailed report. CPs can call in to the system using a toll-free number, identify themselves based on a PIN, and receive information about the patient’s most recent IVR assessment.

CPs and any in-home caregivers in the intervention arm receive informational support for their interactions with each other (Table 3), including materials designed to encourage effective communication regarding the patient’s post-discharge care via principals of motivational interviewing [66]. The CP is asked to initiate 10 minute-15 minute conversations at least weekly with the patient to review the patient’s upcoming post-discharge appointments, self-management goals, and most recent automated assessment reports. During those conversations, CPs are asked to address each of the four pillars of effective transitions within the Coleman model [65,67,68]. CPs and other caregivers can opt to receive a supplemental study training DVD that reiterates and extends the content provided in written materials regarding issues such as role expectations for patients and caregivers, and the suggested structure for the CP-patient follow-up conversations. CPs are encouraged to speak regularly with any in-home caregivers. Effective communication among all caregivers is promoted through communication of “core values” for the program, including: equal access to information and strategies for assigning tasks and resolving potential disagreements. Email summaries following patients’ assessment calls also are sent to patients and other caregivers at their request. CPs and in-home caregivers have access to a toll-free number that they can call to hear automated information including the recommendations from the patient’s most recent assessment. Informational messages in the emails to CPs and study material provided at enrollment emphasize the importance of keeping all caregivers informed.

Individual patient assessment calls include reminders to patients regarding the importance of contacting their care manager if their health deteriorates, and patients can access contact information for their primary provider during each call. CPs are instructed to encourage their patient-partner to contact their clinicians directly, rather than having the CP serve as a communication intermediary.

## Description of measures

Patients complete a telephone interview with a trained research assistant at baseline, 30 days and 90 days post-discharge (Table 4). The goals of the survey are to measure patients’ health service use, health-related quality of life, self-care behaviors, understanding of the transition process, interaction with CPs, and socioeconomic vulnerabilities. At follow-up, patients are asked whether they attended post-discharge appointments and the reasons for any missed appointments. Patients report their pattern of CP telephone and in-person contact. Patients also report information about discussions with their CP regarding their self-care, whether they are comfortable disclosing information to the CP, and whether the CP is too burdened by other life issues to be of much help [69]. Finally, patients report the extent to which they discuss with their CP self-care issues that are the focus of the intervention, including medication use, appointment adherence, and behavioral goal setting. Patients in the intervention group also complete a brief survey of their intervention satisfaction.

CP surveys are conducted at baseline and 90 days via email with follow-up postal mailings. CPs report their pattern of patient contact and the extent to which they discuss with the patient self-care issues that are the focus of the intervention, including use of positive feedback, overcoming barriers to self-care, and reminders about medication refills. CPs’ perceptions of the quality of their relationship with the patient are measured using items similar to those described above for the patient survey [69]. We use second-person adapted versions of the patient measures of adherence and HRQL to assess caregiver’s perceptions of the patient’s status. We use these data to test the hypothesis that the gap between patient and CP perceptions will decrease in the intervention arm of the study, while remaining the same (or even growing) in the control arm.

Semi-structured interviews are conducted with a purposive sample of patients, CPs, in-home caregivers, and clinicians involved in these patients’ transition care. Interviewees are selected so that the sample is diverse in terms of patients’ race, gender, age, and the patient’s baseline health-related quality of life scores. Topics covered during the patient interviews include: patients’ self-efficacy for managing their self-care, the perceived role of their CPs in their transition care, and (for patients in the intervention arm) strengths and weaknesses of the intervention and suggestions for improvement. Similar topics are explored in the CP and clinician interviews, with an added emphasis on possible changes in caregiving strain, and communication with their patient-partner and other caregivers.

Information on patients’ inpatient admissions and outpatient visits to primary care and specialty care is obtained from electronic clinical and billing records. At enrollment, patients are asked to approve retrieval of discharge information from non-affiliated hospitals. Vital status of patients who cannot be contacted at follow-up is determined using requests for information from the health systems, informal caregivers, and a National Death Index search. The IVR calling system automatically captures information on: the time, date, and outcome of all patient call attempts; all patient reported information from the assessments; copies of all CP and care manager e-mails; copies of all care manager faxes, and a record of all CP and care manager log-ins.

### Data management and analysis plan

Patients are the unit of randomization and analysis. Potential variation in outcomes across care managers will be treated as fixed effects. Our primary outcome is 30-day rehospitalization [86,87]. Power for the trial was calculated assuming 22% of usual care patients will experience a rehospitalization within 30 days post-discharge, a rate consistent with prior studies and our own hospital tracking systems [17,88,89]. We calculated the sample size of 760 subjects to provide 80% power to detect a 35% reduction in this rate, assuming a two-tailed alpha of 0.05. To conservatively allow for up to 10% attrition, we will recruit a total of 846 patients. A sample of this size also will be sufficient to detect a medium/small effect on SF-12 scores (i.e., the effect observed in the Sisk [86] trial) and other continuous outcomes.

We will examine differences across treatment groups in baseline measures of study endpoints as well as other potential prognostic indicators, such as patients’ age, race, and gender. As in our prior studies [52,53] we will monitor the completion rates for automated assessment calls and the correlates of system use. Intervention satisfaction ratings will be correlated with system use, patients’ baseline characteristics, and changes in patients’ status between baseline and follow-up.

All outcome analyses will be conducted based on intention to treat. We will use Kaplan-Meier survival curves to assess differences in event-free survival during the 30 and 90 days post-discharge period. Survival curves will be compared using log-rank tests. If chance differences occur between baseline characteristics of CarePartner and usual care groups, we will use Cox proportional hazards models to control for these differences. We will use t-tests to evaluate differences across groups in HRQL change scores between baseline versus 30 days and 90 days. Differences with p<0.05 will be considered statistically significant. In the event that groups are found to be different on important baseline characteristics, we will use MANCOVA models comparing change scores controlling for unbalanced covariates. We expect that most continuous endpoints for the trial such as adherence scores and patients’ satisfaction with the quality of the transition process will be normally distributed. All of these analyses will be based on similar models, with scores at 30 days and 90 days as dependent variables, scores on the same variable at baseline as a covariate, and treatment group as the independent variable.

We will use standard techniques [90] to evaluate changes in the magnitude of the relationship between experimental condition and the outcome before and after potential mediators (e.g., changes in CP-patient communication) are introduced. Analyses of effect moderation will focus on patients’ baseline need for social support, baseline health status, characteristics of their CP (e.g., competing demands), and the presence of an in-home caregiver [91]. We will examine potential intervention effects within subgroups of patients who do and do-not report an in-home caregiver. We then will create multivariate models that include a main intervention effect along with a term for the interaction between intervention group and in-home caregiver availability. We will examine both positive and negative impacts of the intervention on caregiving stress, how CPs and other caregivers distribute responsibilities, and the role of CPs in supporting patients’ interaction with their healthcare team.

## Results

The intervention protocol was approved by the University of Michigan Institutional Review Board, the Ann Arbor VA Human Subjects Committee, and the MidMichigan Health System Institutional Review Board. The trial has been registered in clinicaltrials.gov (ID#NCT01672385). All patients provide written informed consent. A total of 352 patients have been recruited as of August 1st, 2015. Recruitment and data collection are ongoing.

## Discussion

Patients with complex chronic conditions experience frequent and costly hospitalizations [3], and many have unsuccessful transitions back to the community post-discharge [92]. Proactive, post-discharge follow-up can reduce patients’ rehospitalization risk [14,93], but most health systems lack the staffing and information infrastructure to provide these services effectively. New models of transition support articulate the characteristics of effective services [26], but clinicians often cannot provide the intensive self-care assistance that these programs prescribe [94]. Informal caregivers represent a low-cost and effective adjunct to care management [95,96], but caregivers lack: the tools they need to monitor patients’ status, the education necessary to understand patients’ self-care needs, and the skills to know how to respond when issues arise during a transition from hospital to home. Increasingly, high-risk patients live alone; and when spousal caregivers are available, they often are overwhelmed by competing demands, including their own healthcare needs [97,98]. The challenge for improving post-discharge outcomes is to identify services that can support patients, their clinical teams, as well as informal caregivers to improve transition quality while preventing caregiver strain.

In this trial, we are evaluating a unique combination of accessible health technologies and coordinated communication among patients’ informal caregivers and their clinical teams to fill the growing gap between what discharged patients’ need and available resources. A unique advance in the intervention is the explicit provision of transition support not only for patients but also for an informal caregiver or CarePartner. Unlike prior efforts that require additional staffing, the proposed program is designed to enhance care and outcomes using resources that can be easily integrated with available discharge planning procedures. To our knowledge, this is the only study of its kind to challenge the paradigm of clinician-centered post-discharge care by explicitly providing informal caregivers with a structured role and the tools they need to be integral members of the care-support team. Unlike most transition programs that focus on problems related to a single disease, this intervention recognizes that patients frequently are readmitted for any number of health issues other than a given index condition, and therefore the intervention is designed to accommodate patients with a wide range of admitting and discharge diagnoses.

One potential disadvantage of this intervention is that it theoretically could generate unnecessary health service use among patients by increasing their overall sensitivity to relatively benign/self-limiting symptoms. However, data from our prior studies [54–57] have not shown increased outpatient visit rates. The weekly calls, which begin after two weeks of daily calls and two weeks of 3x/week calls, may be insufficient to promptly identify some rapid medical deteriorations. No hard evidence has established the most appropriate frequency of assessments for patients after a hospitalization, and we believe that the proposed plan provides a balance between sensitivity to developing problems and potential false-positives from self-limiting conditions. Patients in the intervention have established access to a care manager and their CP should their symptoms deteriorate between assessment calls. Study participants receive follow-up from a limited number of care managers, which may both limit generalizability and make it impossible to evaluate variation associated with care manager practice style. Finally, baseline surveys with CPs are conducted after patients are randomized. While this is not ideal, we believe that it is more important to initiate patients’ IVR assessments immediately after their discharge so that the first few days following discharge are closely monitored for short-term complications.

In closing, we are testing a unique combination of accessible health technologies and coordinated communication for patients, their informal caregivers, and their clinical teams. A unique advance in this intervention is that-by giving caregivers greater information, education, and support-the program may decrease the burden, stress, and frustration that caregivers frequently experience. We hope that this and similar interventions will address the substantial gap between what discharged patients need and the resources that are realistically available.

## Figures and Tables

**Table 1:** Mapping of intervention components and BOOST program goals.

BOOST goal	Direct support for patients	Informal caregiver support	Support for clinicians
Medication Self-Management	IVR assessments of adherence problems and automated, tailored reinforcement of adherence.	Email, web-based, and IVR feedback about the patient’s adherence problems.	Fax and web-based feed**back about adherence problems.**
Patient-Centered Record (PHR)	PHR provided at discharge. IVR messages refer to the record and reinforce its use with clinicians.	Feedback reports organized according to changes in the content of the PHR. Caregivers can print-out PHRs via the patient’s website.	Access to web-based reports of information in the PHR and reported by the patient during IVR calls.
Follow-up (FU)	FU visits scheduled prior to discharge. Visit information and reminders via IVR calls.	Patients’ visit information reinforced via: email, IVR reminder messages, and the patient webpage.	Fax feedback about serious problems and barriers attending FU visits.
Red Flags (signs and symptoms of a concerning change in health status)	General and disease-specific red flags monitored via IVR calls along with targeted education.	Education about red flags and updates on the patient’s status via email, web, and phone reports.	Fax alerts regarding red flags sent to the care manager immediate following IVR reports.

**Table 2:** Intervention components.

**Patient**
Discharge planning, scheduled follow-up visits and materials based on “4 Pillars of Effective Transitions”
Automated telephonic assessments with feedback on reported problems plus behavioral reinforcement
Structured reminders regarding outpatient follow-up visits with primary care and specialty care
Guidelines for calls with CPs. Access to the website and email reports if they have an Internet connection
DVD describing the program, goals of transition care, and how to organize communication with CPs
**CarePartners**
Summary email and automated telephone reports with structured, tailored advice after each patient automated assessment call including information about how to respond effectively to problems
Access to a web portal with reports on the patient’s status and information about follow-up visits
Automated alert calls for urgent health problems
Web-based information about effective transition support
Guidelines for structured follow-up phone calls with the patient based on IVR assessment results
DVD describing the program, goals of transition care, and how to organize communication with the patient
**Care Manager**
Access to a web portal with summary data on patients’ status
Fax and email alerts for urgent health problems

**Table 3:** Informal caregiver roles and responsibilities.

CarePartner	Opportunities for Participation by Other Caregivers
Review structured emails and web-based reports with feedback based on the patient’s IVR assessments.	Review email summaries and web-based reports if internet access is available.
Engage in structured conversations with the patient based on the assessment results and reinforcing the goals of the transition care.	Follow-up conversations with the patient to reinforce conversations and activities of the CarePartner.
Solicit support from other caregivers for specific patient needs.	As coordinated by the CP, reinforce self-care goals, assist with administrative tasks such as visit scheduling, remind the patient regarding self-care goals, provide emotional support, and gather additional information.
Use the voicemail service to access up-to-date information about the patient’s status if email is unavailable.	Use the voicemail service to access up-to-date information about the patient’s status if email is unavailable.
Understand written materials and DVD provided about the transition process and the patient’s condition.	Understand written materials and DVD provided about the transition process and the patient’s condition.

**Table 4:** Survey-based process and outcome measures.

Domain measures	Patient (PT) and CarePartner (CP) Surveys			References
	Baseline	30 days	90 days	
**Aim 1**				
Inpatient, Outpt, and ED Use	PT	PT	PT	[70,71]
PT Quality of Life: SF-12	PT, CP	PT	PT, CP	[72]
Mortality			CP	
Depression: CES-D	PT, CP	PT	PT, CP	[73]
**Aim 2**				
Care Transition Quality	PT	PT	PT	[74]
Self-Care Behavior				
Self-Efficacy for Self-Care	PT	PT	PT	[75,76]
Medication Adherence	PT, CP	PT	PT, CP	[77]
Medication Beliefs	PT, CP	PT	PT, CP	[78]
Satisfaction with Rx info	PT	PT	PT	[79]
**Aim 3**				
Interaction with CPs and Clinicians				
Social Support	PT, CP	PT	PT, CP	[64,80–82]
Relationship Quality with CP	PT, CP	PT	PT, CP	
Interaction with Care Manager	PT	PT	PT	
CP Burden	CP	PT	CP	[83]
Frequency of Pt-CP Contacts	PT, CP	PT	PT, CP	
Overall Satisfaction with Care	PT	PT	PT	[84]
Program Satisfaction	PT, CP	PT	PT, CP	
Potential Moderators				
Social support, SES, and Health Literacy	PT			[85]
Major diagnoses	PT			
CP health and demographics	CP			
